# Efficacy of a Digital Health Preventive Intervention for Adolescents With HIV or Sexually Transmitted Infections and Substance Use Disorder: Protocol for a Randomized Controlled Trial

**DOI:** 10.2196/47216

**Published:** 2024-02-19

**Authors:** David Cordova, José A Bauermeister, Sydni Warner, The Youth Leadership Council, Patricia Wells, Jennifer MacLeod, Torsten B Neilands, Frania Mendoza Lua, Jorge Delva, Kathryn Bondy Fessler, Versell Smith Jr, Sarah Khreizat, Cherrie Boyer

**Affiliations:** 1 School of Social Work University of Michigan Ann Arbor, MI United States; 2 School of Nursing University of Pennsylvania Philadelphia, PA United States; 3 School of Public Health University of Michigan Ann Arbor, MI United States; 4 The Corner Health Center Ypsilanti, MI United States; 5 Livingston Physician Organization Livingston, MI United States; 6 School of Medicine University of California San Francisco San Francisco, CA United States; 7 Crown Family School of Social Work, Policy, and Practice University of Chicago Chicago, IL United States; 8 School of Social Work Boston University Boston, MA United States

**Keywords:** youth, mHealth, HIV, STI, illicit drugs, primary care, prevention, public health, USA, teens, drugs, drug use, sex, racial minority, risk behavior, engagement, tool, substance use disorder

## Abstract

**Background:**

HIV or sexually transmitted infections remain a significant public health concern in the United States, with adolescents affected disproportionately. Adolescents engage in HIV/STI risk behaviors, including drug use and condomless sex, which increase the risk for HIV/STIs. At-risk adolescents, many of whom are racial minorities, experience HIV/STI disparities. Although at-risk adolescents are disproportionately affected by HIV/STI risk behaviors and infections and although the Centers for Disease Control and Prevention recommends routine HIV/STI testing for adolescents, relatively few adolescents report having ever been tested for HIV/STI. With expected increases in health clinic visits as a result of the Affordable Care Act combined with technological advances, health clinics and mobile health (mHealth), including apps, provide innovative contexts and tools to engage at-risk adolescents in HIV/STI prevention programs. Yet, there is a dearth of efficacious mHealth interventions in health clinics to prevent and reduce both condomless sex and drug use and increase HIV/STI testing for at-risk adolescents.

**Objective:**

To address this gap in knowledge, we developed a theory-driven, culturally congruent mHealth intervention (hereon referred to as S4E [Storytelling 4 Empowerment]) that has demonstrated feasibility and acceptability in a clinical setting. The next step is to examine the preliminary efficacy of S4E on adolescent HIV/STI testing and risk behaviors. This goal will be accomplished by 2 aims: the first aim is to develop a cross-platform and universal version of S4E. The cross-platform and universal version of S4E will be compatible with both iOS and Android operating systems and multiple mobile devices, aimed at providing adolescents with ongoing access to the intervention once they leave the clinic, and the second aim is to evaluate the preliminary efficacy of S4E, relative to usual care control condition, in preventing or reducing drug use and condomless sex and increasing HIV/STI testing in a clinical sample of at-risk adolescents aged 14-21 years living in Southeast Michigan.

**Methods:**

In this study, 100 adolescents recruited from a youth-centered community health clinic will be randomized via blocked randomization with random sequences of block sizes to one of the 2 conditions: S4E mHealth intervention or usual care. Theory-driven and culturally congruent, S4E is an mHealth adaptation of face-to-face storytelling for empowerment, which is registered with the Substance Abuse and Mental Health Services Administration's National Registry of Evidence-Based Programs and Practices.

**Results:**

This paper describes the protocol of our study. The recruitment began on May 1, 2018. This study was registered on December 11, 2017, in ClinicalTrials.gov. All participants have been recruited. Data analysis will be complete by the end of March 2024, with study findings available by December 2024.

**Conclusions:**

This study has the potential to improve public health by preventing HIV/STI and substance use disorders.

**Trial Registration:**

ClinicalTrials.gov NCT03368456; https://clinicaltrials.gov/study/NCT03368456

**International Registered Report Identifier (IRRID):**

DERR1-10.2196/47216

## Introduction

### Background

HIV infections or sexually transmitted infections (STIs) and drug abuse remain significant public health priorities in the United States, with youths being affected disproportionately. Youths aged 15-24 years constitute only 25% of the sexually experienced population, but account for 46% and 50% of HIV infections and new STIs, respectively [1,2]. National surveillance data indicate that youths disproportionately engage in HIV/STI risk behaviors, including condomless sex [3] and licit and illicit drug use [4], which increase their risk for HIV/STI. Despite the disproportionately high rates of HIV/STI and risk behaviors in youths, less than 14% report having ever been tested for HIV infection [3]. Further, many youths are not routinely screened for asymptomatic STIs as recommended by the Centers for Disease Control and Prevention [5]. In Southeast Michigan (the area targeted in this proposed research), HIV/STI cases are disproportionately high [6,7]. To address these significant public health concerns, we developed an innovative mobile health (mHealth) intervention—the practice and dissemination of public health through mobile devices—for health clinic settings. Using this AIDS-Science Track Award for Research Transition (A-START) mechanism, we propose to examine the preliminary efficacy of our mHealth intervention.

The efficacy rates of mHealth [8-10] and brief interventions delivered in health care settings [11-14] aimed at preventing or reducing condomless sex and drug use in youths have been mixed. We believe our intervention, Storytelling 4 Empowerment (S4E), shows promise for several reasons. First, we developed S4E through a community-university collaboration, integrating community-based participatory research principles [15] with the National Institute on Drug Abuse’s prevention guidelines [16]. This process transformed the effective S4E program [17] into an mHealth app [18-22].

S4E is culturally congruent and was adapted in consultation with youths and clinicians from a targeted youth-centered community health clinic. This app is grounded in empowerment [23,24] and ecodevelopmental [22,25-27] theories. S4E uses innovative storytelling scenarios to address key aspects of our intervention’s mechanisms of change, including self-efficacy for condom use and drug use refusal skills (hereon referred to as self-efficacy) and improving clinician-youth communication during the health care visit. These elements aim to increase HIV testing and prevent or reduce condomless sex and drug use behaviors among at-risk youths. Our formative research with youths (defined hereon as adolescents and young adults aged 14-21 years) and clinicians demonstrated that youths in the targeted health care clinic (1) routinely visit the clinic for reproductive and other health care, (2) are at increased risk of HIV/STIs, and (3) found S4E to be feasible and acceptable but also want access to S4E outside of the health clinic to continue participating in intervention activities [18-21,28-30].

Youth-centered community health clinics are an ideal setting for delivering and evaluating the efficacy of an mHealth HIV/STI and drug abuse preventive intervention. Many youths do not seek such services in public health clinics or AIDS service organizations [31]. Furthermore, many primary care pediatric practices do not routinely screen youths for HIV/STIs or drug use [32]. Thus, a gap in knowledge persists regarding efficacious mHealth interventions that improve HIV/STI testing and prevent or reduce condomless sex and drug use in at-risk youths in health care settings [10,18,33]. Since S4E has demonstrated feasibility or acceptability [20], the next important step is to develop a more accessible version of S4E and conduct a stage 1 randomized controlled trial (RCT) [34,35] to examine the preliminary efficacy of S4E in at-risk youths.

### Aims

The proposed study seeks to accomplish the following 2 aims:

1. The first aim is to develop a cross-platform and universal version of S4E. The cross-platform and universal version of S4E will be compatible with both iOS and Android operating systems and multiple mobile devices aimed at providing adolescents with ongoing access to the intervention once they leave the clinic.
2. The second aim is to evaluate the preliminary efficacy of S4E, relative to usual care, to improve HIV/STI testing and reduce HIV/STI risk behaviors in a clinical sample (N=100) of at-risk youths aged 14-21 years living in Southeast Michigan. We will conduct a stage 1 RCT [34,35] to examine the preliminary efficacy of S4E, relative to usual care, in a sample of 100 at-risk youths for 6 months. Our primary outcome is adolescent HIV and STI testing. Secondary outcomes include condomless sex and drug use at 3 months and 6 months postbaseline. As a secondary exploratory aim, we will examine the extent to which our theoretically guided mechanisms of change (ie, self-efficacy, clinician-youth communication) lead to increased HIV and STI testing and prevent or reduce HIV/STI risk behaviors.

The proposed study is innovative, as it is the first to combine mHealth and storytelling to facilitate clinician-youth communication, deliver prevention services, linkage to care, and treatment immediately [27,36,37]. This research program focuses on increasing HIV and STI testing and reducing key risk behaviors such as condomless sex and drug use among at-risk youths. This aligns with the National Institutes of Health HIV/AIDS research priorities [38]. By doing so, it addresses 2 critical goals: (1) preventing and reducing new HIV infections and (2) diminishing HIV-related health disparities. These objectives are among the top 4 priorities outlined in the United States National HIV/AIDS strategy [39].

### Significance

HIV/STI risk behaviors among youths remain the major public health concerns. National surveillance data show that 40.9% of the youths reported condomless sex in the last sexual intercourse [3]. Beyond condomless sex, youths engage in drug use behaviors that increase their risk for HIV/STI. National surveillance data indicate that 66% and 49.1% of youths report lifetime licit and illicit drug use, respectively [4]. Alcohol is the most widely used licit drug, with 37.4% and 66% of youths reporting current and lifetime use, respectively [3,4]. Parallel data from Monitoring the Future study indicate that, from 2008 to 2011, youths’ lifetime, annual, and 30-day prevalence of any illicit drug use have increased [4]. Marijuana remains the most widely used illicit drug with 21.2% and 44% of adolescents reporting current and lifetime marijuana use, respectively [4]. Although the Centers for Disease Control and Prevention recommends HIV/STI testing among youths as part of routine care, many are only being tested based on their perceived risk [5]. HIV/STI testing has important prevention implications, including linkage to both care (eg, preventing transmission of HIV/STI) and important preventive services to remain HIV/STI-free [5].

Racial or ethnic minority youths experience HIV/STI disparities. Youths aged 15-24 years represent 25% of the sexually experienced population and comprise nearly 46% and 50% of HIV infections and new STIs, respectively [1,2]. In 2014, an estimated 9731 youths were diagnosed with HIV in the United States; 78% of these diagnoses occurred in Black/Latino youths [40]. Although the majority of these infections are among young men who have sex with men [40], African American young women are disproportionately affected by STIs, which increases the risk of HIV infection [41]. Given their needs, our sample will consist of a predominantly racial or ethnic minority sample of adolescents in the age group of 14-21 years. This age group spans a time of limited HIV testing and increased HIV/STI risk-taking [3,4] and thereby permits us to intervene at a developmental moment of increased risk [42,43].

Youth in the targeted clinic, many of whom are racial or ethnic minorities, are at disproportionate risk of HIV/STI. Our research shows that, relative to the general US adolescent population, youths in the targeted clinic are more likely to report condomless sex in the last sexual intercourse (40.9% vs 57.9%) and lifetime alcohol (66.2% vs 71.4%) and marijuana (40.7% vs 46.7%) use, respectively [3,20]. Given that condomless sex and drug use are risk behaviors for HIV/STI, not surprisingly, youths in the targeted clinic experience HIV/STI disparities. In the first quarter of 2016 (January-March), 52% (33/63) of youths who received STI testing services tested positive for an STI compared to 25% of the sexually active US youth population [2].

Self-efficacy and clinician-youth communication are potential mechanisms by which change can occur. Researchers have identified a number of etiological factors that shape youth HIV/STI testing [44-46] and risk behaviors [47-49], including intrapersonal (eg, self-efficacy) and ecological (eg, clinician communication) [42,50]. At the intrapersonal level, for example, higher levels of refusal skills and knowledge of self-efficacy increase HIV/STI testing and prevent HIV risk behaviors in youths [42-45,51,52]. At the ecological level, effective sexual communication can ameliorate HIV/STI testing and risk behaviors. Indeed, higher levels of clinician-patient communication have been shown to yield better health outcomes [53-55]. Drawing from this basic science, the proposed study posits that interventions targeting these potential underlying mechanisms of change (ie, improving refusal skills, knowledge, and HIV communication, which in turn will improve self-efficacy and clinician-youth communication) may increase HIV/STI testing and prevent or reduce HIV and STI risk behaviors. Furthermore, understanding the role of etiological factors on youth HIV/STI testing and risk behaviors should be viewed through a cultural and developmental lens [26,56,57]. Therefore, integrating cultural and developmental perspective into theoretical frameworks is important to improve HIV/STI testing and reduce risk behaviors [26,56-58].

Empowerment and ecodevelopmental theories provide a framework for targeting the theoretical underpinnings of our intervention’s mechanisms of change. The empowerment and ecodevelopmental frameworks guide the theoretically driven components of our intervention. The empowerment framework is concerned with linking youths’ strengths and proactive behaviors to helping systems [24,25]. Thus, empowerment-informed interventions seek to enhance the knowledge of the risk factors, refusal communication skills, perceptions of self-efficacy, and engage health care clinicians as resources to accomplish these health goals [24,25]. Equally important is to consider ecological factors. The ecodevelopmental theory [59,60] posits that youths are embedded in integrated ecological systems (microsystem, mesosystem, exosystem, and macrosystem), including developmental and social interaction, which influence and are influenced by the youth [59,60]. In the proposed research, we focus on the health clinic microsystem and limit the conversation to that high-impact system. Microsystems are defined as systems in which the youths participate directly [59,60]. Researchers have extensively applied the ecodevelopmental theory to the family microsystem aimed at improving parent-youth communication [61-63]. With the passing of the Affordable Care Act, we have an opportunity to apply the ecodevelopmental theory to the health clinic microsystem—a system that will only grow larger and increasingly important as a result of the Affordable Care Act—to understand clinician-youth communication [18-21]. Our formative research supports the use of empowerment and ecodevelopmental theories and provides a robust framework for the proposed research.

Health clinic HIV/STI preventive interventions are needed but limited in availability. A recent review of brief (<60 minutes) health clinic interventions examined 31 trials designed to increase HIV/STI testing and prevent HIV/STI risk behaviors. Of these, only 1 study focused on HIV/STI risk behavior outcomes through clinician-youth communication. Findings suggest that, relative to usual care, an audiotaped risk assessment and education intervention showed an increase in communication with providers on STIs and condom use [64]. Findings on the efficacy of brief interventions in health care settings on youth licit and illicit drug use have been mixed [11,14,65]. For example, a recent review of brief interventions in health care settings with clinician approaches was conducted, with 10 interventions designed to prevent or reduce youth alcohol use examined. Of these, only 3 studies were RCTs: 1 had a primarily racial minority sample and 1 was found to be efficacious in reducing alcohol use [11]. Our community-university approach aims to overcome the limitations of these studies. By engaging the community in the development of our mHealth intervention, we believe we will have a greater impact, given that community-university approaches increase uptake and optimize outcomes of prevention programs [66]. Although federal recommendations highlight the need for clinicians to provide youth HIV/STI and drug use preventive services [67,68], efficacious brief interventions designed to increase HIV/STI testing and prevent or reduce condomless sex and drug use are limited [18,33,64,69]. Our formative research suggests that youths in the targeted clinic routinely show for health care visits, with over 550 monthly visits by youths aged 12-21 years. This provides us with an exciting opportunity to impact HIV/STI testing and behaviors through an innovative and interactive mHealth app.

mHealth apps provide new opportunities for engaging youths in preventive interventions, but research is limited. Advances in technology and increased availability provide novel opportunities for prevention scientists [70,71]. Although mHealth interventions are efficacious for adolescent health behaviors [71-73], relatively few studies on HIV/STI testing and risk behaviors exist. A recent systematic review of mHealth apps aimed at addressing the HIV continuum of care identified only 4 published studies and 14 studies underway [10]. Of these, only 7 studies focused on primary prevention of HIV/STI and 6 studies on HIV/STI testing [10]. A limitation of these studies, however, is that they do not target at-risk youths in health care clinics. Our proposed research aims to harness the widespread use of mobile technology [74] and deliver and evaluate an mHealth HIV/STI intervention among at-risk youths, many of whom are racial minorities, in a health care clinic.

Storytelling for Empowerment (SFE) is an effective face-to-face intervention to translate and test a new mHealth preventive intervention. Registered with Substance Abuse and Mental Health Services Administration’s National Registry of Evidence-Based Programs and Practices, SFE aims to increase self-efficacy and communication about HIV risk behaviors and has been shown to prevent or reduce HIV/STI risk behaviors and increase (1) HIV/STI and drug use prevention knowledge, (2) HIV/STI communication, and (3) perception of harm and self-efficacy in refusing drugs [17,75,76]. Adapting SFE into an mHealth version for health care clinics was ideal for several reasons, including SFE has already demonstrated efficacy with other youth populations and the use of storytelling makes it highly flexible and easily transportable into a brief mHealth modality. Based on empowerment [24,25] and ecodevelopmental [22,26,77] frameworks, the mHealth version of SFE, S4E, consists of 3 modules targeting youth: (1) HIV/STI risk assessment, (2) HIV/STI, and (3) alcohol/drugs. The risk assessment assesses youth HIV/STI risk behaviors. Both the HIV/STI and drug modules consist of videos (developed from focus group data on community-specific epidemiology) focused on HIV/STI testing and risk and promotive behaviors, knowledge development, interactive activities, and messaging aimed at increasing clinician-youth communication.

Despite federal guidelines urging clinicians to provide youth HIV and drug use preventative care [67,68], our research [19,20] and that of others [78-80] demonstrate that clinicians’ limited HIV/STI communication training and embarrassment to discuss HIV/STI risk pose as challenges to engage youths in these conversations. With funding from the National Institute of Mental Health (R25MH067127), we developed a theory-driven and culturally congruent clinician component to overcome these barriers, which provides clinicians with (1) youth risk assessment scores, (2) tailored HIV/STI communication interviewing toolkits (eg, reflective questioning), and (3) tailored resources to link youths with care. We recognize that youths in health clinics represent one segment of the youth population, but nonetheless an important segment at disproportionate risk of HIV/STI [20,28].

### Preliminary Studies

#### Study 1: Feasibility and Acceptability of S4E

##### Study Design

Youths (n=30) were recruited from Southeast Michigan and were primarily African Americans (20/30, 67%) and females (22/30, 73%) with a mean age of 16.23 (SD 2.09) years. We used a community-engaged research approach with 3 phases, that is, formative focus groups (n=29), app development, and feasibility testing (n=30). We used agile software development [81]. Formative focus group data collection and app development occurred simultaneously.

##### Results

**Figure 1:**
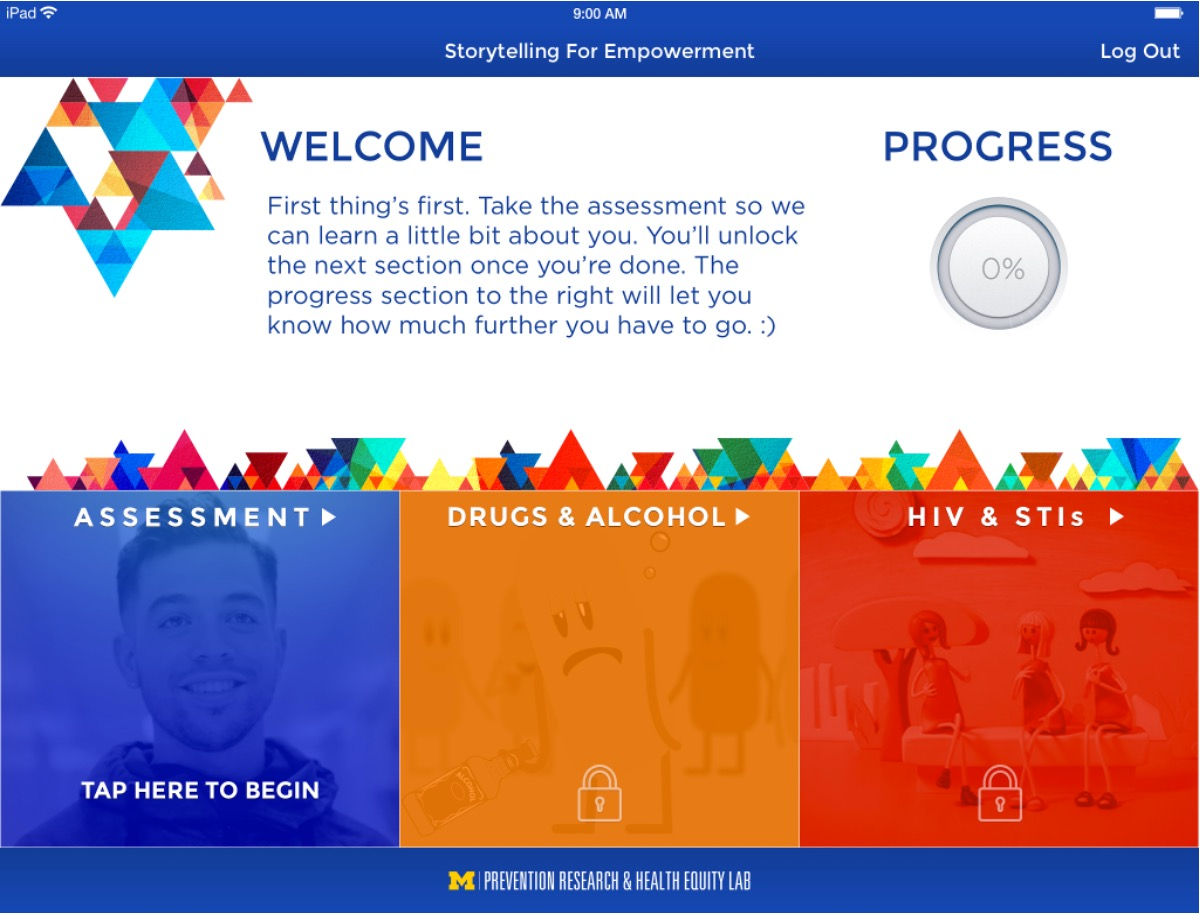
A theory-driven and culturally congruent mobile health version of Storytelling for Empowerment.

We developed a theory-driven and culturally congruent mHealth version of SFE (Figure 1). Both qualitative and quantitative data from the Session Evaluation Form [82] (mean 1.42, SD 0.46) and Client Satisfaction Questionnaire [83] (mean 3.46, SD 0.47) indicate that S4E was acceptable to youths. Data also showed that youths regularly visit the clinic (ie, 550 visits per month), visit the clinic multiple times (mean 3 visits), and are at increased risk of HIV/STI risk behaviors. Since we demonstrated the feasibility, the next important step was to conduct a stage 1 preliminary efficacy RCT of S4E on adolescent HIV/STI testing and risk behaviors [18-21].

#### Study 2: Development of S4E Clinician App

Employing Community-Based Participatory Action Research principles, we collaborated with clinicians from the targeted youth-centered community health clinic in Southeast Michigan (NIMH R25MH067127) to inform the development of the clinician components for the S4E app [84]. We used agile software development [81] for the creation of prototype models, obtained rapid feedback from clinicians regarding user interface/experience, and a feedback loop for revisions and to finalize the app. The S4E clinician app provides clinicians with (1) youths’ risk assessment scores, (2) tailored HIV/STI communication interviewing toolkit (eg, reflective questioning, positive reinforcement), and (3) tailored resources to link youths with care. Feasibility or acceptability testing is underway and study findings will be incorporated into this proposed A-START research.

## Methods

### Study Design

The first aim is to develop a cross-platform and universal version of S4E. The purpose of developing a cross-platform and universal version of S4E is to create a more accessible app that is compatible with different operating systems (ie, Android and iOS) and multiple mobile devices, thereby providing youths with ongoing access to the intervention outside of the clinic. Finalizing the universal version of S4E will be streamlined because the app developer, The Annex Group, created S4E for the iOS operating system to use on iPads, which will serve as an existing framework (eg, code, design database). Similar to the methodology employed in our formative research, as part of the iterative process, we will hold weekly meetings with The Annex Group to discuss all aspects of finalizing the app, including framework and user interface or user experience. At the completion of the first aim, we will have finalized S4E, which will be used for our stage 1 preliminary efficacy RCT in the second aim. We will pilot test all study procedures prior to addressing the second aim.

The second aim is to evaluate the preliminary efficacy of S4E to improve HIV/STI testing and reduce HIV/STI risk behaviors in a clinical sample (N=100) of at-risk adolescents aged 14-21 years living in Southeast Michigan. To test the preliminary efficacy of S4E, we will conduct a stage 1 RCT and use a mixed between/within-subjects design with 2 levels of intervention (S4E and usual care) as the between-subjects factor and 3 repeated measures assessments (baseline, 3-, and 6-months postbaseline) as the within-subjects factor.

### Participants

A clinical sample of 100 youths and clinicians (n=6) will be recruited from Corner Health Center. In 2015, the clinic reported over 5600 visits from individuals aged 12-21 years; therefore, recruiting 100 youths will be feasible. With respect to participant race, ethnicity, and age, we will recruit a sample that is representative of the clinical population. As in our previous studies [28-30], we expect the majority of participants to be racial minority youths and be sexually active (50/70, 71% report past 90-day oral, vaginal, or anal sexual intercourse) [20,28].

### Inclusion Criteria

Participants must be female or male youths aged 14-21 years, sexually active, live in Southeast Michigan, and have access to a smartphone or tablet (51/70, 73% report having access to smartphone [85]). Youths must see an enrolled clinician to participate in this study. Exclusion criteria include report of prior psychiatric hospitalization by the adolescent, visible cognitive impairment due to drug use, and adolescent reports (tentative or firm) plans to move out of the Southeast Michigan area during the study.

### Recruitment

A multipronged recruitment strategy will be implemented: face-to-face interactions, flyer distribution, engagement in the clinic’s waiting area, and informing youths with upcoming clinic appointments at the clinic about the study. Potential participants will be informed about the study by research staff, with details about its voluntary nature and the RCT design. Those interested will be sent a study web-based app link, where they can provide digital consent and screen for eligibility. Participants aged 13-17 years will be given a waiver of parental permission as per Michigan regulations. Following consent, participants will undergo baseline assessment and randomization through the study’s web-based app. They will then be informed of their group allocation. Research staff will introduce participants to the app and ensure proper navigation. Participants will be incentivized, receiving a total of US $120: US $30 at baseline, US $40 at the 3-month follow-up, and US $50 at the 6-month follow-up.

### Retention

To prevent attrition, we will ask youths to provide the names and contact information of 3 persons who will always know where they can be reached. These names will help maintain contact with the youths in case they move or their telephone lines become disconnected. Youths may choose to provide the names and contact information of individuals they trust, such as a primary caregiver, relative, or significant adult figure. Youths will be informed that if the research team cannot reach them and needs to contact these individuals, the team will only communicate their intention to contact the youths about a health study. Additionally, a sample of 6 clinicians will be recruited. Similar to our formative research, research staff will provide an overview of the study to all clinicians during a staff meeting, and they will be informed that participation is voluntary. To prevent coercion of clinician participation by clinic administration, potential participants will not need to go through nor inform the clinic administration for participation. An initial list of potential clinician participants will be developed, and research staff will follow up with those clinicians who express interest in this study.

### Clinicians

Clinicians will be assigned to either S4E or control condition. In lieu of providing each clinician with an incentive for their participation in the study, the clinic will receive US $2000, which will benefit the entire clinic. Randomization will occur after baseline assessment. Three of the 6 clinicians will be assigned to the S4E condition and trained according to research criteria. Clinicians will receive a 1-hour training, encompassing content delivery, app navigation, risk assessment viewing, note-taking within the app, and effective communication strategies for discussing substance use and sexual behaviors. A review training will ensure adherence to the protocols. All clinicians who express interest in this study and do not report (tentative or firm) plans to move out of the region during this study will be eligible to participate.

### Experimental and Control Conditions

Participants were assessed at baseline, randomized to intervention (n=50) or control (n=50) groups using block randomization [86], and then reassessed immediately postintervention, at 3 months, and at 6 months. Participants in the S4E condition will initially engage with the intervention by using iPads available in the waiting area. This includes iPads allocated for this study and an additional 10 from the principal investigator’s pilot studies. They will also be instructed on how to download the intervention app onto their personal devices, enabling continued participation in intervention activities after leaving the clinic.

### S4E Description

Informed by our formative research, the intervention lasts approximately 60 minutes and was found to be feasible and acceptable to youths. Content includes the theoretically driven components of SFE [17,75,76]: (1) storytelling scenarios, (2) drug use and HIV/STI knowledge development, (3) interactive activities, (4) increasing self-efficacy to prevent or reduce sexual risk and drug use behaviors and increase HIV/STI testing, (5) clinician-youth communication, and (6) highlighting prevention principles (Textbox 1 and Textbox 2).

**Experimental condition of S4E intervention.**

**Adolescent components**

- Risk assessment: Youths complete an HIV/sexually transmitted infection (STI) risk behavior assessment, which include items from the CRAFFT (Car, Relax, Alone, Forget, Friends, Trouble) [87] measure, HIV testing, and sexual and drug use behaviors, and provide opportunity or receptivity to being counselled (1 min to complete).
- HIV/STI module: Youths are exposed to storytelling scenarios, including community-specific HIV/STI epidemiology (eg, risk, protective behaviors). Following the videos, youths operate an app aimed at increasing condom use self-efficacy, HIV/STI knowledge development, and interactive activities aimed at engaging youths and testing knowledge. Additionally, youths receive messaging aimed at facilitating clinician-youth communication and HIV/STI testing (30 min to complete).
- Alcohol/drugs module: Adolescents are exposed to storytelling scenarios, including community-specific alcohol/drug epidemiology (eg, prevalent licit and illicit drugs). Following the videos, adolescents operate an app aimed at increasing drug use refusal self-efficacy, drug use knowledge development, and interactive activities aimed at engaging youths and testing knowledge. Additionally, adolescents receive messaging aimed at facilitating clinician-adolescent communication (30 min to complete).

**Clinician components**

- Risk assessment scores: Clinicians are provided youth risk assessment scores, identifying at-risk youths, and provided opportunity to reinforce information provided to youths through the modules (above).
- Tailored HIV/STI communication toolkit: Based on empowerment and ecodevelopmental theories, clinicians are provided a communication toolkit, which includes examples of open-ended reflective questioning, positive reinforcement statements, HIV/STI risk probing, and empowerment messaging.
- Tailored resources and referrals: Clinicians are provided tailored community-identified local resources and referrals, including HIV/STI testing and linkage to care.

**Underlying mechanisms of change.**

Consistent with empowerment and ecodevelopmental theories, Storytelling 4 Empowerment provides an opportunity to be counselled, which in turn:

- Increases clinician-youth communication
- Increases engagement of health care clinicians as collaborators to address HIV/sexually transmitted infection (STI) and drug use concerns
- Increases clinician-youth sexual risk communication
- Increases HIV/STI knowledge
- Increases condom use
- Increases condomless sex refusal skills
- Increases clinician-youth drug use communication
- Increases drug use knowledge
- Increases refusal skills
- Enables clinicians reinforce prevention strategies and link youths to HIV/STI testing and linkage to care

### Control Condition

Participants in usual care (ie, control condition) will not receive the S4E intervention from the study staff. The clinic’s usual care includes a standard risk behaviors intake form, pamphlets highlighting resources, and reproductive and health care services.

### Measures

#### Youth HIV/STI Testing and Risk Behaviors

After the intervention, we will assess whether youths requested to receive HIV/STI testing at the clinic, and at 3 months, and 6-months postbaseline (yes/no). Adolescent unsafe sexual behavior will be measured (timepoints 1-3) by using items extracted from the Sexual Behavior Instrument [88]. This gated instrument will assess the adolescent’s past 90-day condom use, number of sexual partners, and contraceptive use (not condoms). This measure also assesses the existence of an STI during their lifetime and in the past 90 days. Licit and illicit drug use behaviors will be assessed (timepoints 1-3) using items from the Monitoring the Future study [4]. Youths will be asked whether they have used licit or illicit drugs in their lifetime and in the past 90 days. Youths who report “Yes” to past 90-day sex will be asked to report frequency of drug use prior to sex. These measures have been used in our formative research [18-21].

#### Potential Mediators: Clinician-Youth Communication and Self-Efficacy

Completed by both the clinicians (α=.70) and youths (α=.69), clinician-youth communication will be assessed (timepoints 1-3) using items adapted from the Matched Pair Instrument (19 items) [89]. The Matched Pair Instrument assesses the process and content of communication, including verbal and action-related behaviors performed by clinicians [89]. Responses range from “1=strongly disagree” to “5=strongly agree” on a 5-point Likert scale. A sample statement for clinicians and youths is, “Encouraged the patient/me to express his or her/my thoughts concerning drug use behaviors.” Youths’ self-efficacy will be assessed (timepoints 1-3) using 2 scales, namely, the Condom Self-Efficacy Scale (19 items, α=.85) [90] and Drug Use Resistance Self-Efficacy (24 items, α=.98) [91]. Responses range from “1=not sure at all” to “4=definitely sure” on a 4-point Likert scale. A sample question for the youth is, “How sure are you that you can refuse if a friend offers you marijuana at a party and you do not want it?” Additionally, youths and clinicians will respond to a demographic survey, wherein they will have to fill in the details of their date of birth, gender identity, sexual orientation, age, ethnicity or race, income, and education.

### Intervention Dosage for Both Clinicians and Youths (Not Given to Participants)

S4E includes a login procedure. In addition to the login procedure serving as a mechanism by which the secure access and confidentiality of participants is ensured, it will facilitate the close monitoring of participants’ dosage. The login procedure will be used to record whom (ie, participant), when (ie, day/time), how long (eg, dosage), and for what purpose (eg, module). We will assess participants’ satisfaction with S4E across platforms (eg, mobile phone) and the use of the intervention once they leave the clinic. Higher frequencies of access and longer durations of app use will indicate higher levels of dosage and engagement with the intervention.

### Statistical Analysis

Given the sample size and stage 1 RCT pilot nature of our study, we will not conduct a formal test of efficacy. Researchers affirm [92] that effect size estimates obtained using stage 1 RCT data may not be reliable, given their large variability. Therefore, our primary purpose was to estimate the critical parameters [92] required to inform the potential effects of S4E in a stage 2 RCT. As part of the preliminary efficacy process, we expect the following intervention exposure relative to usual care participants:

1. Hypothesis 1: S4E participants will have increased odds of repeat HIV/STI testing postintervention.
2. Hypothesis 2: S4E participants will have decreased odds of past 90-day condomless sex at 3 months and 6 months postbaseline.
3. Hypothesis 3: S4E participants will have decreased odds of past 90-day licit and illicit drug use at 3 months and 6 months postbaseline.

**Figure 2:**
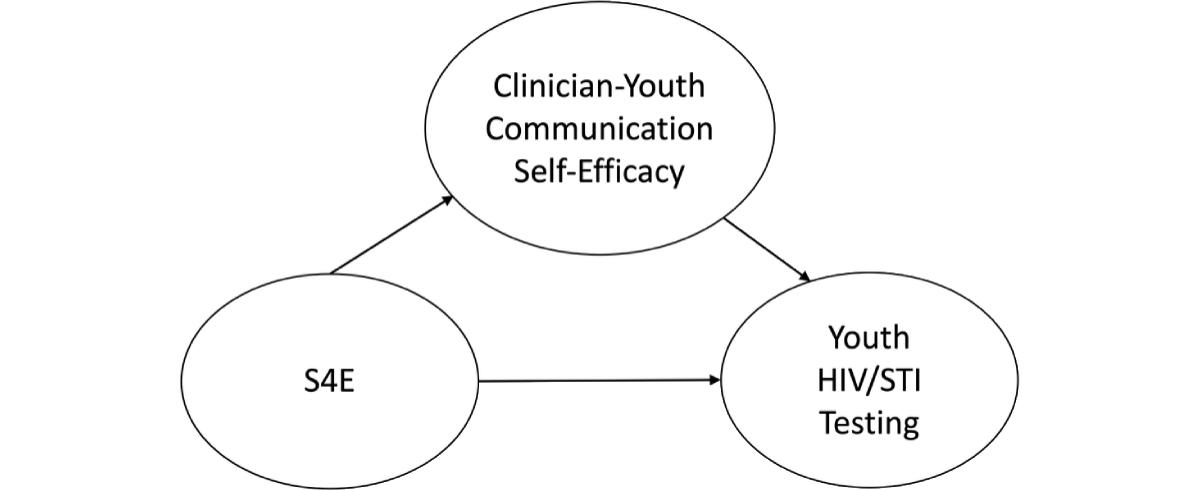
Potential mechanisms of change caused by Storytelling 4 Empowerment. S4E: Storytelling 4 Empowerment; STI: sexually transmitted infection.

As a secondary exploratory aim to examine potential mechanisms of change (Figure 2), we also anticipate that S4E participants compared to usual care participants will report (1) higher mean levels of clinician-youth communication and (2) drug use and HIV/STI self-efficacy.

To test differences between conditions postrandomization, we will plot means and proportions by condition over time for descriptive analysis of overall patterns of change across time in the outcomes for the S4E and usual care conditions. We will use linear mixed models (LMMs) for continuous outcomes (eg, clinician-adolescent communication scale scores measured to address exploratory aim) and generalized LMMs (GLMMs) for discrete data (eg, HIV/STI testing measured to address H1) to evaluate the proposed preliminary hypotheses. GLMMs fitted to discrete outcomes (eg, condomless sex) will employ a binomial distribution with a logit link; GLMMs fitted to count outcomes will use the best-fitting distribution from the Poisson family (eg, zero-inflated Poisson) with the log link function [93]. We will assess whether clustering effects associated with clinic physicians must be accounted for in these analyses. All mixed models will be estimated via maximum likelihood estimation and will be fitted to ensure that all requisite information is available in the survey and data to perform the types of analyses typically undertaken in a stage 2 RCT. Similarly, although the modest sample size precludes investigating mediation and moderation formally, we will employ the same LMM and GLMM approaches described above to examine potential mediators (ie, clinician-adolescent communication, self-efficacy) and moderators (eg, gender, race) of the S4E intervention.

### Power Analyses

Due to the modest sample size, significance testing will be de-emphasized. The purpose of stage 1 RCT is to determine preliminary efficacy rather than to conduct formal hypothesis tests; nevertheless, we conducted power analyses using nQuery Advisor version 7.0 [94] to estimate the magnitude of effect we could observe, given our pilot sample size. With 50 cases per group, we would have 80% power to detect an odds ratio of 3.7 in receiving HIV testing immediately postintervention between the 2 conditions. This would be considered a large effect size [95]. Using GLMM models to compare the 2 groups in terms of trends in the probability of binary outcomes over 6 months (eg, condomless sex), we performed a custom simulation study to estimate the size of the interaction between group × time that we could detect with 80% power when fitting our models (and assuming a within-subject correlation of 0.1 in the binary measures). We would be able to detect percentages of 70% in the intervention group at 3 months and 25% at 6 months as representing a significantly different reduction in the percentage with this outcome over time (ie, a significant group × time interaction) with approximately 80% power, which would again be considered a large effect.

### Ethics Approval

The principal investigator (DC) received approval (HUM00158089) from the University of Michigan institutional review board to begin research in February 2017 and was awarded funding from the National Institute on Drug Abuse on February 01, 2017.

## Results

This study has been designed to develop an mHealth intervention program (S4E) and evaluate its preliminary efficacy to improve HIV/STI testing and reduce HIV/STI risk behaviors among youth populations. Our study findings will contribute to reducing HIV/STIs and risk behaviors among youths. The development of the intervention has been completed, and recruitment for the preliminary efficacy trial began in May 2018. We completed the trial in August 2020. We recruited 100 participants, data analyses are underway, and the results are expected to be published by December 2024.

## Discussion

The overarching goal of this program of research is to move a program of intervention research from efficacy to scale and to examine the extent to which these modules are generalizable to similar youth populations. If found to have preliminary efficacy, the next step in this program of research is to conduct a stage 2 RCT to examine the effects of S4E on youth HIV/STI testing and risk behaviors. We are aware that control group participants may unintentionally receive the experimental group content. Although it might not be possible for youths to see the same clinician at 3 months and 6 months postbaseline, we have the Corner Health’s support that youths will only see clinicians in the condition to which they are assigned (ie, S4E or usual care). The proposed age range (14-21 years) may seem wide; however, this is an age group at increased risk for HIV/STI [1,2]. Further, this age range was established in consultation with Corner Health who, in considering both the strengths and limitations, preferred a universal app that was relevant to the clinical population. Our 2-arm, baseline, 3-month, and 6-month postbaseline design was chosen, given the scope of the R03 A-START mechanism.

Our protocol explores the behavioral change practice methods (ie, proposed mechanisms underlying the observed changes), namely, clinician-youth communication and self-efficacy. The use of storytelling scenarios, created by youths through a community-engaged research method grounded in community-based participatory research principles, could offer an innovative strategy for future studies. This approach highlights the importance of the relationship between clinicians and youths as well as the role of self-efficacy.

Our protocol may benefit the society by providing compelling evidence for the preliminary efficacy of an mHealth intervention in promoting HIV and STI testing and reducing sexual and substance use risks among adolescents and young adults. The promising intervention, combined with the proposed recruitment and retention strategies, may provide evidence for larger scale trials. Given the pressing demand for efficacious interventions in this domain, this protocol may have a significant societal impact. Further, this proposed research aligns well with the broader goals of the National Institutes of Health HIV/AIDS research priorities [38], the National HIV/AIDS strategy [4], and the recommendations issued by the US Preventive Services Task Force to reduce youth’s HIV and drug use risks by linking them to screening and care services in community health clinic settings [67,68].

## Abbreviations

A-START: AIDS-Science Track Award for Research Transition
GLMM: generalized linear mixed model
LMM: linear mixed model
mHealth: mobile health
RCT: randomized controlled trial
S4E: Storytelling 4 Empowerment
SFE: Storytelling for Empowerment
STI: sexually transmitted infection
